# On the Blood-Globules, Their Formation and Use

**Published:** 1842-12

**Authors:** 


*On the Blood-Globules, their Formation and their Use.*—M. Donne recognizes three kinds of particles in the blood,—red globules, or blood-globules, properly so called, white globules, and globulines of chyle, as he calls them. The red globules are flat in all kinds of blood; circular in the mammalia; elliptical in fishes, reptiles, and birds. The elliptical globules alone have a solid substance in their interior: no similar appearance has he been able to detect in the circular globules. Water, he says, renders the flat blood-globules spherical; and acetic acid dissolves completely the round globules of the mammalia, but leaves an insoluble portion in the elliptical globules of birds, reptiles, and fishes. This insoluble portion, he says, is the central nucleus. He therefore regards the blood-globule as composed of a flattened vesicle containing a solid nucleus in the elliptical globules, and a fluid only in the circular ones. The elliptical blood-globule of the camel he found no exception to the general rule that those of the mammalia have no central nucleus.

The white globules he describes as colorless, irregular in their contour, and having a granular aspect, and exist in the blood of all animals. Water breaks them up, and he imagines them to be composed of a cyst containing three or four solid granules.

The globulines again he describes as extremely small granules similar to those of the chyle.

M. Donné gives the following theory of the mode of formation and uses of these different parts. The globulines are the product of the chyle, which is continually being added to the blood. Three or four of these unite together, and whilst circulating with the blood, receive an albuminous envelope. They thus form the white globules. The white globules once formed change little by little their forms; become flattened, colored; the granular matter in their interior becomes homogeneous or dissolved, and they are thus transformed into the red or proper blood-globules. The blood-globules themselves have only a passing existence; they dissolve after a certain time, and constitute the so-called *liquor sanguinis*. He says that certain substances, as milk, are capable of being immediately transformed into blood-globules by being ejected into the blood-vessels; and he regards the spleen as the organ more especially charged with the important function of the manufacture of blood-globules. He adds, that a minute examination of the vascular tissues shows, that at no point do the blood-globules leave their vessels to assist in the formation of the organs of the body, or to unite with other organic elements. Hence, he infers, it must be the fluid part of the blood which transudes through the vascular walls, and that this part must be the fluid essentially concerned in the process of organization.—*Edin. Med. and Surg. Jour.*, July 1842, from *Comptes Rendus*, March 1842.

